# Nurse practitioners and physician assistants working in ambulance care: A systematic review

**DOI:** 10.12688/f1000research.25891.1

**Published:** 2020-09-29

**Authors:** Risco van Vliet, Remco Ebben, Nicolette Diets, Thomas Pelgrim, Jorik Loef, Lilian Vloet

**Affiliations:** 1 Emergency Medical Service, RAV Brabant MWN, 's-Hertogenbosch, Brabant, 5212VM, The Netherlands; 2 Research Department of Emergency and Critical Care, HAN University of Applied Science, School of Health Studies, Nijmegen, Gelderland, 6525 EJ, The Netherlands; 3 Emergency medical service RAVU, Utrecht, Utrecht, 3723 BC, The Netherlands; 4 IQ healthcare, Radboud Institute for Health Sciences, Radboud university medical center, Nijmegen, Gelderland, 6500 HB, The Netherlands

**Keywords:** nurse-practitioners, physician-assistants, ambulance care, patient outcomes, implementation, emergency medical services

## Abstract

**Background**: This review aims to describe the activities of nurse practitioners (NPs) and physician assistants (PAs) working in ambulance care, and the effect of these activities on patient outcomes, process of care, provider outcomes, and costs.

**Methods**: PubMed, MEDLINE (EBSCO), EMBASE (OVID), Web of Science, the Cochrane Library (Cochrane Database of Systematic Review), CINAHL Plus, and the reference lists of the included articles were systematically searched in November 2019. All types of peer-reviewed designs on the three topics were included. Pairs of independent reviewers performed the selection process, the quality assessment, and the data extraction.

**Results**: Four studies of moderate to poor quality were included. Activities in medical, communication and collaboration skills were found. The effects of these activities were found in process of care and resource use outcomes, focusing on non-conveyance rates, referral and consultation, on-scene time, or follow-up contact

**Conclusion**s: This review shows that there is limited evidence on activities of NPs and PAs in ambulance care. Results show that NPs and PAs in ambulance care perform activities that can be categorized into the Canadian Medical Education Directives for Specialists (CanMED) roles of Medical Expert, Communicator, and Collaborator. The effects of NPs and PAs are minimally reported in relation to process of care and resource use, focusing on non-conveyance rates, referral and consultation, on-scene time, or follow-up contact. No evidence on patient outcomes of the substitution of NPs and PAs in ambulance care exists.

**PROSPERO registration**: CRD42017067505 (07/07/2017)

## Background

Ambulance utilisation has increased in the Western world over the past 20 years, potentially compromising access, quality, safety, and patient outcomes ^1, 2^. Population ageing, changes in social support and accessibility, increasing community health awareness, patients presenting themselves with higher complexity and comorbidities, repeated ambulance care requests, and ambulance care request for primary healthcare problems have been described as associated factors for this increase ^1, 3– 6^. The pressure to reduce costs and the potentially negative effects of this increase of ambulance utilisation have led to the redefinition of the roles of professionals in prehospital care ^1, 2^. With the impending rise in demand for health services, an effective utilization of the workforce is paramount to ensure high-quality yet cost-effective health service delivery ^7^. This can be done by optimising triage and ambulance allocation, but also by introducing other types of healthcare professionals in the ambulance domain. A possible solution to improve the balance between the increasing demand for care and the decreasing supply of medical healthcare workers is enhancing the role of allied healthcare workers, such as nurse practitioners (NPs) or physician assistants (PAs) ^8 ^.

The first NPs and PAs in the Dutch healthcare system made their appearance in 2001 and 2004. NPs are situated in the nursing domain and perform broadening activities in the medical domain within selected groups of patients and simultaneously on deepening activities in the nursing domain. PAs focus on broadening and deepening activities in the medical domain, within their medical specialty.

Several reviews about the implementation of NPs and PAs have been performed ^9– 12^. These reviews have revealed not only a higher quality of care but also an increase in patient satisfaction and that NPs and PAs have the potential to reduce doctors’ workloads and direct health care costs. However, this research has been limited to long-term care facilities and primary health care; there currently is no evidence pertaining to what activities NPs or PAs in ambulance care perform and what the effects of these activities are.

## Aims

Therefore, this review has two aims. First, to describe the activities of nurse practitioners and physician assistants working in ambulance care. Second to describe the effects of these activities on patient outcomes, process of care, provider outcomes, and costs.

## Methods

### Design

This study is a systematic literature review reported according to the steps of Cochrane Handbook ^13^ and reported to conform with the Preferred Reporting Items for Systematic Reviews and Meta-Analysis (PRISMA) statement. For background and an extensive method section, see the study protocol ^14^.

### Search strategy

The Cochrane Database for Systematic Reviews and PROSPERO were inspected for similar reviews or protocols. No (pending) review was identified, so systematic searches were performed in PubMed, MEDLINE (EBSCO), EMBASE (OVID), Web of Science, the Cochrane Library (Cochrane Database of Systematic Reviews), and CINAHL Plus in November 2019.

Search strategies were developed to represent terms for ambulance care and NPs or PAs. Full search strategies per database are provided as extended data ^15^.

### Study selection procedure

Searches were not restricted by year of publication. All types of peer-reviewed systematic reviews, and quantitative or qualitative designs in real clinical practice or simulation situations on NPs or PAs working in ambulance care for all kinds of patients were included. Conference abstracts, narrative reviews, editorials, personal communications, and unpublished studies were excluded.

Studies were included if they (a) described activities of professionals with a master’s degree in ambulance care (NPs or PAs) and/or described the effects of the NP or PA on patient outcomes, process of care, provider outcomes and costs.

Due to the heterogeneity of the names that are used for the emergency medical service (EMS) professional worldwide ^16^, we began with a broad search. Some terms covered a variety of different professionals; for example, the education level of the emergency care practitioner (ECP) can be that of a paramedic or a nurse. We explicitly searched for professionals with a master’s degree and excluded studies where this was not present.

Two reviewers (RvV and ND) independently screened the title and abstract of each potentially relevant study. Differences between the reviewers were resolved through discussion. Couples of two independent reviewers (RvV, RE, ND, JL, and LV) screened the full texts of the remaining articles. In addition, two reviewers (RvV and RE) screened the reference lists of the included articles.

### Quality assessment

To assess the quality of observational studies, we used a tool developed for evaluating primary research papers in a variety of fields ^17^. Couples of independent researchers (RvV, RE, ND, JL, and LV) performed this assessment. Differences between two reviewers were resolved through discussion; in cases of doubt, a third reviewer from another couple made the final decision.

### Data extraction, synthesis, and presentation

Couples of independent researchers (RvV, RE, ND, JL, and LV) extracted the data. Due to the heterogeneity of study designs, settings, countries, care providers, interventions, and outcome measures, a meta-analysis was not possible; the results of this systematic review are therefore presented in tabular form.

## Results

### Review statistics

**Figure 1.**
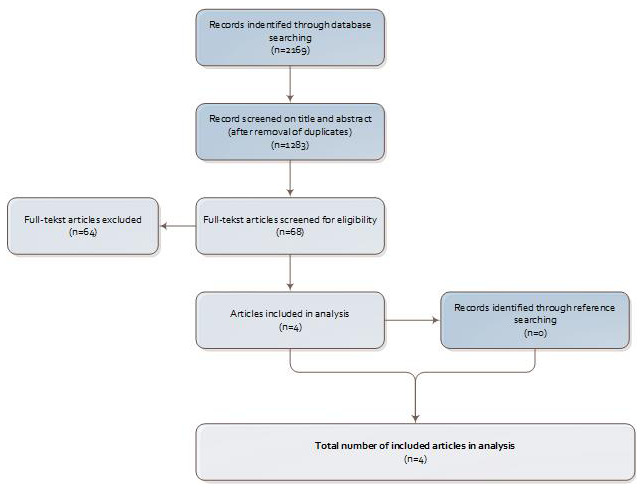
Study selection process.

The initial search identified 1283 unique records; 68 articles were included for full-text screening (see Figure 1), from which we included four articles for data extraction. A list of excluded articles and the reasons for their exclusion ( *n* = 64) is provided as extended data ^18^. Common reasons for exclusion were a non-master educational level, the lack of peer review, and the wrong setting (not prehospital ambulance care).

### Study characteristics ( Table 1)

Designs of the included studies comprised of one cross sectional ^19^, one retrospective observational ^20^, one action research ^21^, and one retrospective descriptive review ^22^ ( Table 1). Two studies were performed in the UK, one in the Netherlands and one in the USA. All these studies extracted the data from ambulance run records or patient records. The focus of three of these studies ^19, 21, 22^ was primarily on ambulance care, where the retrospective observational study ^20^ had a broader perspective of home care, ambulance care, and emergency care. One study ^19^ compared the PA with a registered nurse (RN), two compared the NP with other EMS professionals (e.g., paramedics) ^20, 21^ and one solely described the NP ^22^ without comparison. 

**Table 1.** Characteristics of quantitative included studies (n=4).

1 ^st^ author (year) Country (ref)	Design	Aim	Setting	Data source	Patients (n)	Professionals (n)
Bloemhoff (2016) NL	Cross-sectional	To provide insight in the outcome of patient care between PA's and RN's, working as solo emergency care providers in EMS	2 EMSs	Ambulance run records (n=972)	Patients with urgency level A1 (arrival <15min) and A2 (arrival <30min) who were treated by Pa (n=493) or RN (n=498)	PA (n=2), RN (n=23)
O'Hara (2011) UK	retrospective observational	To compare the quality and safety of care provided by ECPs and non-ECPs (eg paramedic or NP)	3 Different types of emergency care setting: (1) static centers (ED, walk-in-center, minor injury unit) (2) ambulance/ care home services (Mobile) (3) primary care out of hours services	Patient records (n=480)	Static center (n=318) Ambulance (n=80) Primary care out of hours services (n= 80)	ECPs vs non-ECPs (eg paramedic, NP)
Walsh (2001) UK	Action Research	To provide insight in the proportion of patients that could be treated at the scene by an NP	1 EMS	Patients record (n=130)	Patients (n=130) attended by a NP	NP (n=1)
Sanko (2019) USA	retrospective, descriptive review	describes the initial 18-month experience implementing this new APRU	1 EMS	Patient records (n=812)	812 APRU- attended patients	NP (n=1)

Abbreviations: APRU Advanced Provider Response Unit, ECP Emergency care practitioner, ED Emergency department, EMS Emergency medical services, NP Nurse practitioner, PA Physician assistant, RN Registered nurse

### Quality assessment (extended data ^23^)

The cross sectional study ^19^ is of moderate quality due to the representativeness of results, and small population. The other three studies ^20– 22^ are of poor quality.

***Activities by a NP or PA ( Table 2).*** Two articles reported on activities NPs or PAs perform in ambulance care ^19, 20^, these activities were related to medical skills, communication and collaboration. For medical skills, the usage of the SCEBS methodology and overall care, assessment, investigation, management, and quality of record registration were described ^19, 20^. For communication, the provision of medical advice and for collaboration referral to the ED or GP were described ^19^.

**Table 2.** Activities by a NP or PA.

Data extraction outcomes				
Medical skills	Bloemhoff (2016) NL	Monitoring vital sign of patient by Pa (n=2) RN (n=23) • Respiratory rate • Oxygen saturation • Systolic blood pressure • Diastolic blood pressure • Pulse rate • ECG/ heart rhythm • GCS/AVPU • Glucose • Body temperature • Pain intensity score SCEBS methodology used Systematic physical exams of organ tract systems • Suture • Administer medication not in national EMS standard	PA (n=493) • 294 (60%) • 219 (44%) • 229 (47%) • 229 (7%) • 316 (64%) • 39 (8%) • 430 (87%) • 55 (11%) • 33 (7%) • 0 (0%) • 77 (16%) • 155 (31%) • 16 (3%) • 47 (10%)	RN (n=498) • 343 (69%) • 246 (49%0 • 269 (54%) • 255 (51%) • 325 (65%) • 144 (29%) • 437 (88%) • 95 (19%) • 12 (2%) • 72 (15%) • 0 (0%) • 0 (0%) • 0 (0%) • 0 (0 %)
	O’Hara (2011) UK	Ambulance/ care home service (mobile) Overall care • Mean (SD) • 95% CI (for mean) • Mean difference (95% CI) Assessment • Mean (SD) • 95% CI (for mean) • Mean difference (95% CI) Investigation • Mean (SD) • 95% CI (for mean) • Mean difference (95% CI) Management • Mean (SD) • 95% CI (for mean) • Mean difference (95% CI) Quality of records • Mean (SD) • 95% CI (for mean) • Mean difference (95% CI)	ECP (n=40) 3.7 (1.1) 3.4 to 4.1 -0.45 (-0.97 to 0.73) 3.8 (1.2) 3.4 to 4.2 -0.53 (-1.1 to 0.03) 4.1 (1.4) 3.6 to 4.5 -0.30 (-0.92 to 0.32) 3.8 (1.4) 3.4 to 4.3 -0.43 (-1.02 to 0.17) 4.0 (1.2) 3.6 to 4.4 -0.23 (-0.74 to 0.29)	Non-ECP (n=40) 4.2 (1.30) 3.8 to 4.6 4.3 (1.3) 3.9 to 4.7 4.4 (1.4) 3.9 to 4.8 4.3 (1.3) 3.8 to 4.7 4.2 (1.1) 3.8 to 4.6
Communication	Bloemhoff (2016) NL	Provide the patient of medical advice	PA (n=493) 235 (48%)	RN (n=498) 0 (0%)
Collaboration	Bloemhoff (2016) NL	Referral after EMS treatment on scene In case of referral: type of health care organisation referred to • Referral to GP • Referral to ED	n=489 245 (50%) 44 (18%) 201 (82%)	n=482 351 (73%) 86 (25%) 265 (75%)

Abbreviations: ECG electrocardiograph, ED emergency department, EMS emergency medical services, GCS/AVPU Glasgow coma scale/ Alert Voice Pain Unresponsive, GP general practitioner, SCEBS Somatic complaints, Cognitions, Emotions, Behaviour and Social functioning of the patient, NP nurse practitioner, PA physician assistant

### Effects of activities ( Table 3)

None of the included studies reported on patient outcomes, care provider outcomes, or costs; the studies did report on process of care and resource use.

All four studies ^19– 22^ reported on process of care outcomes. The outcomes used included the proportion of non-conveyance, the number of referrals in non-conveyance patients, the number of consultations, the length of on-scene treatment, the follow-up contact of non-conveyance patients, diagnostic measurements, adherence to guidelines and protocols and, number of performed interventions. One study reported on resource use.

**Table 3.** Process of care and resource use outcomes.

Data extraction outcomes and effect				
Proces of care outcomes	Bloemhoff (2016) NL	Interventions described in the national EMS standard • Administer medication according to national EMS standard • Placement intravenous drip • Supply of oxygen • Immobilisation Interventions not described in the national EMS standard • Suture • Administer medication not in national EMS standard • Medial advice to patient	PA (n=493) 84 (17%) 23 (5%) 14 (3%) 10 (2%) 16 (3%) 47 (10%) 235 (48%)	RN (n=498) 87 (17%) 36 (7%) 5 (1%) 0 (0%) 0 (0%) 0 (0%)
		Length of treatment time on scene median in minutes (IQR) ^b^	n=489 25 (19)	n=489 26(17)
		Referral after EMS treatment on scene In case of referral: type of health care organisation referred to • Referral to GP • Referral to ED	n=489 245 (50%) 44 (18%) 201 (82%)	n=482 351 (73%) 86 (25%) 265 (75%)
		In case of consultation: type of health care professional consulted n % • General practitioner • Emergency department • Other (e.g. medical specialist)	n=164 119 (73%) 18 (11%) 27 (16%)	n=84 73 (87%) 2 (2%) 9 (11%)
		Follow up contact after prehospital EMS care n (%) Within 72 h Within 24 h	n=493 25 (5%) 16 (3%)	n=493 20 (4%) 12 (2%)
		Follow up contact of non-referred patients (to ED/GP) after prehospital EMS care n (%) Within 72 h Within 24 h	n=244 9 (4%) 7 (3%)	n=129 4 (3%) 2 (2%)
	Bloemhoff (2016) NL	Provide the patient of medical advice	PA (n=493) 235 (48%)	RN (n=498) 0 (0%)
	Walsh (2001) UK	Non-conveyance rates	20% treat-and-release when NP was staffed at regular ambulance, 34% treat-and- release when NP was staffed at solo first response vehicle	
	Sanko (2019) USA	Non-conveyance rates	N=400 (50,5%) were treated and released on scene or medically cleared and transported to a mental health urgent care; 49.5% required or ultimately opted for transport by LAFD ambulance to the ED.	
		Length of treatment time on scene	N=400	
		median in minutes (IQR) ^b^	21:47min	
		Safety patient attended by APRU		
		911-patietns non-conveyed.	26 (6.5%) re-contacted 911 within 3 days	
		Deceased after 2 months	n=1 (Gun Shot)	
		Experience of care and perceived quality of care	n=51 96.9% of patients rated their overall quality of care as good, very good or excellent.	
Resource use	Sanko (2019) USA	EMS resources		107 other EMS resources were released from the scene of these mental health incidents and put back in service
			18 high utilizers of 911 were connected with a social work organization, and 12 of 18 (66.7%) decreased their use of EMS in the 90-days following	

Abbreviations: EMS emergency medical services, GP general practitioner,NP nurse practitioner, PA physician assistant, RN registered nurse

***Non-conveyance (n=3).*** Three studies reported on non-conveyance ^19, 21, 22^ and showed non-conveyance rates ranging from 20% –50% for PAs. Non-conveyance rates for the NP were not described.

***Referral and consultation (n=1).*** One study ^19^ found that PAs refer 50% of their patients to another health care professional (e.g., a GP or an emergency department (ED)) while RNs referred 73%. Furthermore, PAs consulted other health care professionals (e.g., a GP, an emergency physician, or a medical specialist) significantly more often compared to RNs.

***On-scene time (n=2).*** One study found no significant difference between PAs and RNs regarding the length of on-scene treatment time ^19^. Another study described ^22^ an average length of treatment time on scene of 21.47min, but made no comparison with other EMS professionals.

***Follow-up contacts (n=1).*** Follow-up contact after the completion of prehospital EMS care also indicated no significant differences between PAs and RNs ^19^.

***Resource use (n=1).*** One study ^22^ found in 107 cases other EMS resources were released from the scene and put back in service while the NP attended the patient, (by default, two units respond to a call). 18 high utilizers of 911 were connected with a social work organization, and 12 of 18 (66.7%) decreased their use of EMS in the 90-days following.

## Discussion

This review aimed to describe which activities NPs or PAs deploy in ambulance care, and if there were effects on patient outcomes, process of care, provider outcomes, and costs.

The results indicate that little is known on the activities PAs and NPs deployed in ambulance care. This can be explained by the relatively young professions these professionals represent. The activities that were identified can be categorized using the Canadian Medical Education Directives for Specialists (CanMED) framework. The CanMEDS system is a widely used instrument to describe medical professionals activities and forms the basis of the education of NPs and PAs ^24^. A competent professional seamlessly integrates all seven competencies CanMEDS roles ^24^ (Medical Expert (the integrating role), Communicator, Collaborator, Leader, Health Advocate, Scholar and Professional). However, the activities found in this review can be categorized into the medical professional, communicator and collaborator. This is remarkable because the full NP and PA profiles includes seven CanMEDS roles. There are several reasons why all seven roles are not reported on. First, it is possible that not all seven roles are applicable in ambulance care, or are not visible in the primary process of ambulance care. Also, PAs and NPs have only recently integrated into the ambulance care system, a clear job description or interpretation of their duties may be lacking. Developing a systematic description of the roles and competences of NPs or PAs in ambulance care would therefore be useful.

Although there are differences in education between NPs and PAs, there also seems to be a large degree of overlap in the tasks that NPs and PAs perform in practice ^25^; for instance, both professionals perform tasks that are part of the medical process, such as, drafting and evaluating treatment plans, and carrying out interventions ^25^.

Results shows that little is known on the effects of the activities of NPs and PAs in ambulance care. Some effects found can be linked to process of care and resource use. We found no effects on patient outcomes or care provider outcomes. Reviews ^12, 25, 26^ in other health care settings revealed an increase in quality of care and patient satisfaction. Evidence in primary care ^26^, elderly care ^3^, and out-of-hours primary care ^27^ suggests that the substitution of NPs or PAs is feasible with at least the maintenance of quality and no increase in costs.

Although we have found no description on the effect on costs, Walsh *et al.* ^21^ assumed that the substitution of NPs could produce substantial savings for the EMS and relieve the burden on hard-pressed ambulance and ED. Bloemhoff *et al.* ^19^ recommended further exploration into the costs.

Further research is necessary to draw any conclusions on the effects of the substitution of NPs and PAs in ambulance care for multiple outcomes. This should be addressed by using the six dimensions of quality of healthcare: 1- effectiveness, 2- efficiency, 3- patient safety, 4- accessibility, 5- timeliness and 6- target population directed ^26^. Measuring these outcomes within all phases of the ambulance process (from initial call, to handover or referral) will gain more insight in the effects of PAs and NPs in ambulance care.

## Strengths and limitations

A strength of this systematic review is that the search began with a broad strategy for six online databases, following the quality standards from the Cochrane Handbook ^13^ and reported to conform with the PRISMA statement ^28^.

One limitation of this review lies in the fact that our broad search strategy produced only four studies that described NPs or PAs working in ambulance care. Within these studies, the settings are completely different which made it impossible to perform a meta-analysis. Due to the diversity of the professionals working in ambulance care worldwide, it was difficult to identify the educational level of the professionals. Another limitation concerns the quality assessment tools for quantitative and qualitative designs a variety of these tools exists without a clear evidence base ^13^.

## Conclusion

This review shows that there is limited evidence on activities of NPs and PAs in ambulance care. Results show that NPs and PAs in ambulance care perform activities that can be categorized into the CanMED roles of Medical Expert, Communicator, and Collaborator. The effects of NPs and PAs are minimally reported in relation to process of care and resource use, focusing on non-conveyance rates, referral and consultation, on-scene time, or follow-up contact. There is no evidence on patient outcomes of the substitution of NPs and PAs in ambulance care. Further research is necessary to provide insight into these effects.

## Data availability

### Underlying data

All data underlying the results are available as part of the article and no additional source data are required.

### Extended data

Figshare: Appendix 1 search strategies.docx. https://doi.org/10.6084/m9.figshare.12949730.v1 ^15^

Figshare: Appendix 2 Reason full text exclusion.docx.

https://doi.org/10.6084/m9.figshare.12949736.v1 ^18^

Figshare: Appendix 3 Quality of quantitative studies (n=4).docx. https://doi.org/10.6084/m9.figshare.12949748.v1 ^23^

### Reporting guidelines

Figshare: PRISMA checklist for ‘Nurse practitioners and physician assistants working in ambulance care: A systematic review’ https://doi.org/10.6084/m9.figshare.12949766.v1 ^29^

Data are available under the terms of the Creative Commons Attribution 4.0 International license (CC-BY 4.0).
