# Supplementary material for: Nurse practitioners and physician assistants working in ambulance care: A systematic review
Source: F1000Res. 2020 Sep 29;9:1182. [Version 1] doi: 10.12688/f1000research.25891.1 (PMC7788519; doi:10.12688/f1000research.25891.1)
Supplement: Peer review history

# Peer review: Reviewer response for version 1

**DOI:** 10.5256/f1000research.28573.r75887

**Published:** 2021-01-06

**Authors:** Barbara Todd

**Affiliations:** 1 Hospital of the University of Pennsylvania, Philadelphia, PA, USA

Thank you for the opportunity to review and comment on this manuscript. As care models continue to emerge, it is improved to report on outcomes of care related to NP and PA provided care. I have a few comments.

1. The abstract could be more robust in the background section to provide the context and necessity for this review.
2. There are limited studies as pointed out by the authors and concern with comparison of PA to RN rather than PA to NP care. I suspect there would be differences dependent on RN scope in some of the regions.
3. Is there enough evidence to put forward a systematic review, obviously more research is needed on PA and NP care in ambulance services.
4. It would have been helpful to describe utilization of NPs and PAs in other care delivery models.
5. What was the acuity of the patients managed by NP and PA, how does that compare to others delivering the same care. I think this would have provided rich context to the manuscript.

Are the rationale for, and objectives of, the Systematic Review clearly stated?

No

Is the statistical analysis and its interpretation appropriate?

Not applicable

Are sufficient details of the methods and analysis provided to allow replication by others?

Yes

Are the conclusions drawn adequately supported by the results presented in the review?

Partly

Reviewer Expertise:

NP and PA models of care

I confirm that I have read this submission and believe that I have an appropriate level of expertise to confirm that it is of an acceptable scientific standard, however I have significant reservations, as outlined above.

# Peer review: Reviewer response for version 1

**DOI:** 10.5256/f1000research.28573.r72764

**Published:** 2020-12-22

**Authors:** Lisette Schoonhoven

**Affiliations:** 1 Nursing Science, Julius Center for Health Sciences and Primary Care, University Medical Center Utrecht, Utrecht University, Utrecht, The Netherlands; 2 School of Health Sciences, Faculty of Environmental and Life Sciences, University of Southampton, Southampton, UK

Thank you for the opportunity to review this manuscript. The topic presented is very timely given the strains healthcare is under and the future developments in the Western population. 

 This review is performed well and the fact that only 4 papers were identified which only reported on limited outcomes signals the need for further research in this area. 

 After reading the paper I do have some questions and comments.

1. The abstract does not state why it is important to do this review. Perhaps this is due to a restriction in number of words.
2. In the background the authors mention broadening activities and deepening activities for NPs and PAs. Examples of these activities could have added clarity for the reader who is not familiar with these positions.
3. The results on effects of the activities seem to focus on a comparison with RN's. While I understand this is the focus of some of the papers included, this is not the focus of this review and does not answer the research question.
4. Given that there are only 4 studies describing NPs and PAs in ambulance care, I wonder if other studies are needed first before focusing on quality outcomes. For example a study on where and how are these professionals are employed and a focus on their CanMEDS roles. Why are these professionals the answer to the problems mentioned in the introduction? The authors could have discussed this more thoroughly. 
5. The limitations mention the quality assessment tools used. This comes as a bit of a surprise. Also, given that only quantitative designs were found (according to table 1) I am not sure how the lack of an evidence base for the tools for assessing qualitative designs can be a  limitation in this paper. 
6. Overall, I wonder if doing a systematic review limited to scientific papers should have been the design of choice for this review. Perhaps also looking at policy documents etc could have provided a richer overview of the positioning of these professionals in the field of ambulance care. This could have been mentioned in the discussion.

Are the rationale for, and objectives of, the Systematic Review clearly stated?

Partly

Is the statistical analysis and its interpretation appropriate?

Not applicable

Are sufficient details of the methods and analysis provided to allow replication by others?

Yes

Are the conclusions drawn adequately supported by the results presented in the review?

Yes

Reviewer Expertise:

Nursing Science

I confirm that I have read this submission and believe that I have an appropriate level of expertise to confirm that it is of an acceptable scientific standard, however I have significant reservations, as outlined above.

# Response

**Published:** 2020-12-23

**Authors:** Risco van Vliet

**Affiliations:** RAV Brabant MWN, The Netherlands

Dear Lisette Schoonhoven

 Thank you for your valuable review of our manuscript and the critical questions you have asked.

 Before we started this review, we searched for available information that provides insight into NPs and PAs working in ambulance care. We found very little and what was written was often a personal story and not judged by a peer reviewer. We wanted to tackle this in a scientific way and expected that little had been written but were surprised by the low inclusions. Because of this, you can indeed question whether this is the correct design to answer this research question. Perhaps we would have emphasized this more strongly in the discussion.

 After evaluating the process and based on the information found, we find a reason to continue scientific research into the role of NPS and PAs in ambulance care and to support initiatives and distant developments with solid research.

# Peer review: Reviewer response for version 1

**DOI:** 10.5256/f1000research.28573.r72226

**Published:** 2020-10-23

**Authors:** Elena Lopatina

**Affiliations:** 1 University of Calgary, Calgary, AB, Canada

Thank you for the opportunity to review this interesting systematic review. The review explores activities of nurse practitioners and physician assistants working in ambulance care and effects of those activities in terms of patient and provider outcomes, processes of care, and costs. The fact that only 4 studies were identified and none of the identified studies reported on patient or care providers outcomes, or costs highlights the need for more research in this area.

 Two minor comments appeared while reviewing the manuscript:

1. Introduction, paragraph 3: NPs’ roles and activities as well as outcomes of those activities have also been explored in the context of inpatient and outpatient specialized care. Also, it would be useful to provide references to showcase previous research on NPs’ and PAs’ activities in various settings. 
2. Authors limited their search to care providers with a Master degree only, thus excluding 22 articles during the full-text review. While authors recognize the existing differences in the level of training of NPs and PAs working in ambulatory care, it would be helpful to explain the rationale for focusing on providers with a Master degree only. Would authors expect roles and activities to differ between providers with a Master degree and without it? Given the low number of articles included and a relatively large number of full-texts excluded based on this criteria, one would wonder if it would be useful to take a broader approach.

Are the rationale for, and objectives of, the Systematic Review clearly stated?

Partly

Is the statistical analysis and its interpretation appropriate?

Not applicable

Are sufficient details of the methods and analysis provided to allow replication by others?

Yes

Are the conclusions drawn adequately supported by the results presented in the review?

Yes

Reviewer Expertise:

Models of care, quality of care, non-physician care providers

I confirm that I have read this submission and believe that I have an appropriate level of expertise to confirm that it is of an acceptable scientific standard.
